# The clinical result of arterialized venous free flaps for the treatment of soft tissue defect of the fingers

**DOI:** 10.1097/MD.0000000000016017

**Published:** 2019-06-07

**Authors:** Malrey Lee, Young-Keun Lee, Dong-Hee Kim

**Affiliations:** a The Research Center for Advanced Image and Information Technology, School of Electronics & Information Engineering, Chonbuk National University; b Department of Orthopedic Surgery, Research Institute of Clinical Medicine of Chonbuk National University – Biomedical Research Institute of Chonbuk National University Hospital, Jeonju, Chonbuk, Republic of Korea.

**Keywords:** arterialized venous free flap, finger, soft tissue injury

## Abstract

The purpose of this study is to report the clinical results of the arterialized venous free flaps in reconstructing soft tissue defects of the finger and to extend the indications for the use of the flaps based on clinical experiences of the authors.

We retrospectively reviewed the records of 35 patients who underwent an arterialized venous free flaps for a finger reconstruction, between May 2007 and August 2015. The mean size of flap was 4.8 ± 1.23 × 3.1 ± 0.84 cm. The donor site was the ipsilateral volar aspect of the distal forearm in all cases. There were 17 (48.6%) cases of venous skin flaps, 9 (25.7%) cases of innervated venous flaps, 7 (20%) cases of tendocu taneous flaps, and 2 (5.7%) case of innervated tendocutaneous flap. The vascularity of recipient beds was good except 8 (22.9%) cases (partial devascularity in 3, more than 50% avascularity [bone cement] in 3, and chronic infected bed in 2).

Of the 35 cases, 29 (82.9%) cases (including 3 cases who had more than 50% avascularity recipient bed) showed complete survival. 3 (8.6%) cases, which had partially devascularity of distal phalanx in recipient bed, showed partial necrosis (*P* = .015). The mean number of included veins was 2.4 ± 0.5 for a flap.

A forearm arterialized venous free flap is a useful procedure for single-stage reconstructing of a soft tissue or combined defect of a finger, we consider that this technique could be applied to fingers despite an avascular or insufficient vascular recipient bed if the periphery of recipient bed vascularity was good and if the recipient beds were free from infection.

## 1 Introduction

With the advances of the concept of perforator flap, the use of conventional flaps such as the free digital, free medial plantar, and free peroneal artery perforator flaps in finger reconstruction has produced good outcomes.^[1–3]^ However, for reconstruction of the injured hand, especially the finger, an arterialized venous free flap (AVF) is still a good operation technique, which has many advantages. It can prevent stiffness of the finger joint and offer thin and pliable flap that fits easily around the contours of the fingers. It also offers 1 stage reconstruction of composite defect with incorporation of tendon, or nerve within the flap with less donor site morbidity. For this reason an AVF has been used in reconstructions in fingers by several authors.^[4–8]^ However, AVFs are not commonly selected as the first choice for microsurgical reconstruction because of the unstable postoperative recovery sequence that involves severe postoperative swelling, discoloration, and bullae formation. Additionally, unpredictable necrosis of the flap, especially in the cases where vascularity of the recipient beds are not good causes many surgeons to hesitate in selecting it because of the fear of the problems which could be occurred by different hemodynamics of flaps as compared with other conventional flap's. To improve the survival rate of flap, we created a design where the center of the flap was placed on the area where the vein distribution was most abundant; a relatively smaller vein was used as the afferent vein and a larger vein was used as the efferent vein. The length of the venous pedicle of the afferent vein should be as short as possible to minimize the number of intravenous valves. Furthermore, we anastomosed the vessels out of the injury zone and attempted to anastomose more efferent veins, but it was not easy to control the number of veins because the flap size was small.

### 1.1 Consent

This study received approval from our institutional review board at Chonbuk National University Hospital (No. 2016-01-001-003).

## 2 Material and method

Thirty-five patients, who underwent an AVFs for a finger reconstruction between May 2007 and August 2015, were reviewed retrospectively. Patients included 31 (89%) men and 4 (11%) women with a mean age of 40.26 ± 10.07 years (range, 22–61 years). The mean follow-up period was 9.17 ± 1.93 months (range, 2–12 months). The causes of soft tissue defects were 27 (77%) machine crushing injury, 3 (8.6%) burns with crushing injury, 3 (8.6%) electrical saw injury, 1 (2.9%) sickle injury, and 1 (2.9%) tube injury. Twenty-four (71%) patients were performed operation within 1 week, other 10 patients were performed operation between 2 and 6 weeks according to the progression of soft tissue necrosis.

### 2.1 Size and composition of the flaps

Flap size was classified according to Woo's classification.^[7]^ Six (17%) flaps were small (<10 cm^2^), 27 (77%) flaps were medium, 2 (6%) flaps were large (>25 cm^2^) (Table 1). Seventeen (49%) were pure cutaneous venous flaps (CVF). There were 18 (51%) cases of compound flaps that included 2 different types of tissue, such as tendon, nerve along with the skin flap. Among these, there were 9 (50%) cases of innervated venous flap (IVF), 7 (39%) cases of tendocutaneous venous flap (TVF), 2 (11%) cases of innervated tendocutaneous flap (Table 1).

**Table 1:**
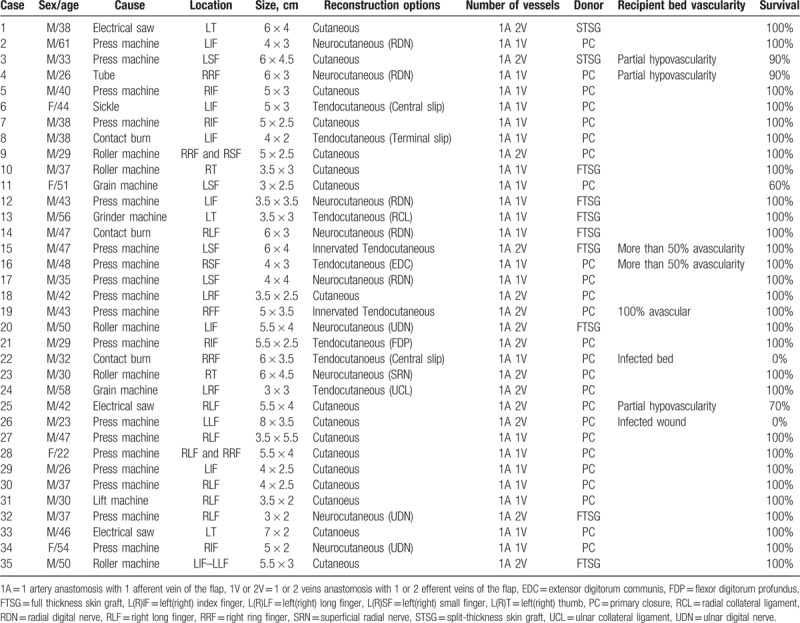
Details of 35 cases of arterialized venous free flap.

### 2.2 Donor and recipient sites

Forearm distal volar side was the donor site in all cases. Recipient sites were single digit in 32 (91.4%) cases, 2-digits in 2 (5.7%) cases and 3-digits in 1 (2.9%) case. Single digit included 5 thumbs, 12 index fingers (IF), 6 long fingers (LF), 4 ring fingers (RF), and 5 small fingers (SF). Two-digits included 1 LF and RF, 1 RF and SF. Three-digits included IF, LF, and RF.

The recipient sites of IVF included 2 radial digital nerve (RDN) reconstruction in IFs, 2 ulnar digital nerve (UDN) reconstruction in IFs, 2 UDN and RDN reconstruction in LFs, 1 RDN reconstruction in RF, 1 RDN reconstruction in SF, 1 superficial radial nerve reconstruction in thumb.

The recipient sites of TVF included 3 extensor tendon reconstruction in IFs, 1 flexor tendon reconstruction in IF, 1 radial collateral ligament (CL) reconstruction of interphalangeal (IP) joint in thumb, 2 extensor tendon reconstruction in SFs, 1 ulnar CL reconstruction of distal interphalangeal (DIP) joint in RF, 1 extensor tendon reconstruction in RF.

The vascularity of the recipient beds was partially devascularity at the distal phalanx in 3 (8.6%) cases, more than 50% avascularity at the bone cement was packed in the bone defect site in 3 (8.6%) cases, and some infection in 2 (5.7%) cases (Table 1).

### 2.3 Surgical technique

All patients underwent surgery under general or regional anesthesia with tourniquet control. First, we debrided the recipient area for the preparation of recipient vessels. We extended the skin incision to the out of the zone of injury, dissected digital artery or common digital artery which did not affect the finger survival and got the veins in the dorsal aspect of finger. At this time we dissected enough to perform the vascular anastomosis out of injury zone. We made a template with surgical glove, which included the site of artery, vein, and contour of defect. The tourniquet was inflated to 100 mm Hg, which allowed the veins to engorge. The veins were marked on the forearm distal volar aspect with a skin marker. To make the center of the flap placed on the area where the vein distribution was most abundant, we applied the already manufactured template on the forearm and drew the flap more larger than the recipient site to prevent postoperative swelling, edema, and tension. At this time the relatively smaller vein was planned to be used as the afferent vein and the larger vein as the efferent vein.

After the afferent vein and efferent vein were dissected long enough, flaps were dissected superior to the muscle fascia, included only skin and subcutaneous veins. In 10 (28.6%) IVFs, during the dissection of pedicles, the distribution of the cutaneous sensory nerve was first checked through a proximal incision of the flap and then harvested as mentioned above. In 9 (25.7%) TVFs, during the flap dissection, we included the palmaris longus (PL) tendon. The average length of transplanted tendon was 3.44 ± 1.1 cm (range, 2–5 cm).

**Figure 1:**
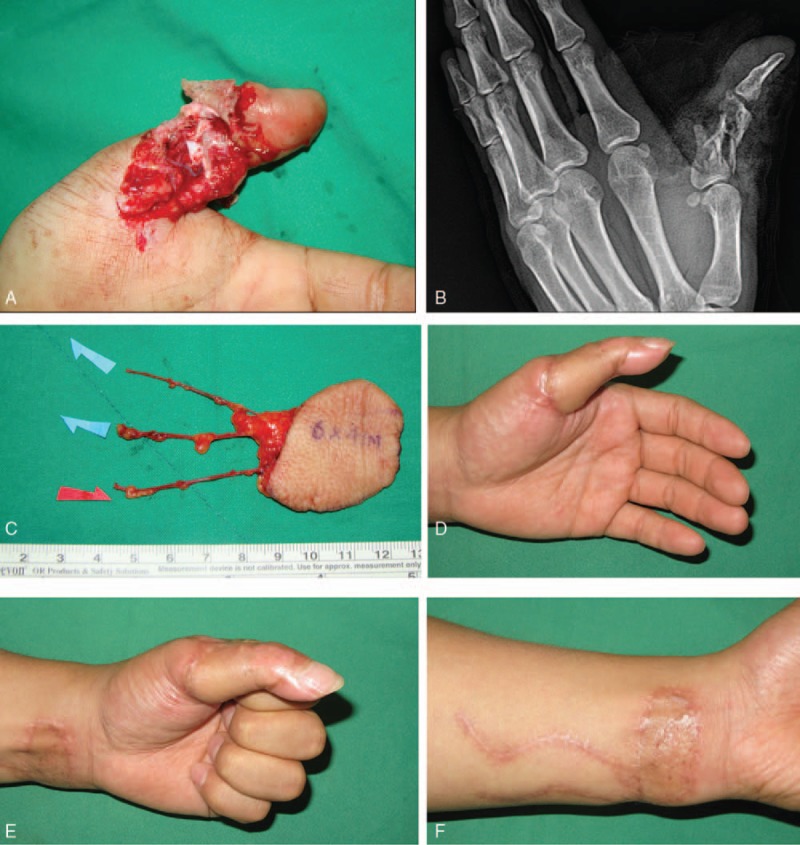
A 38-yr-old man with open fracture of left thumb. (A and B) Preoperative view of left thumb shows crushed open fracture of the proximal phalanx. (C) Dissected venous skin flap about 6 × 4 cm including 3 veins from the volar aspect of the ipsilateral distal forearm. (D–F) Postoperative view of the donor and recipient site 7 mo later, showing that contour is almost normal. Grip and pinch is 35 kg and 9 kg, which is 70% and 65% that of the intact contralateral hand, respectively.

**Figure 2:**
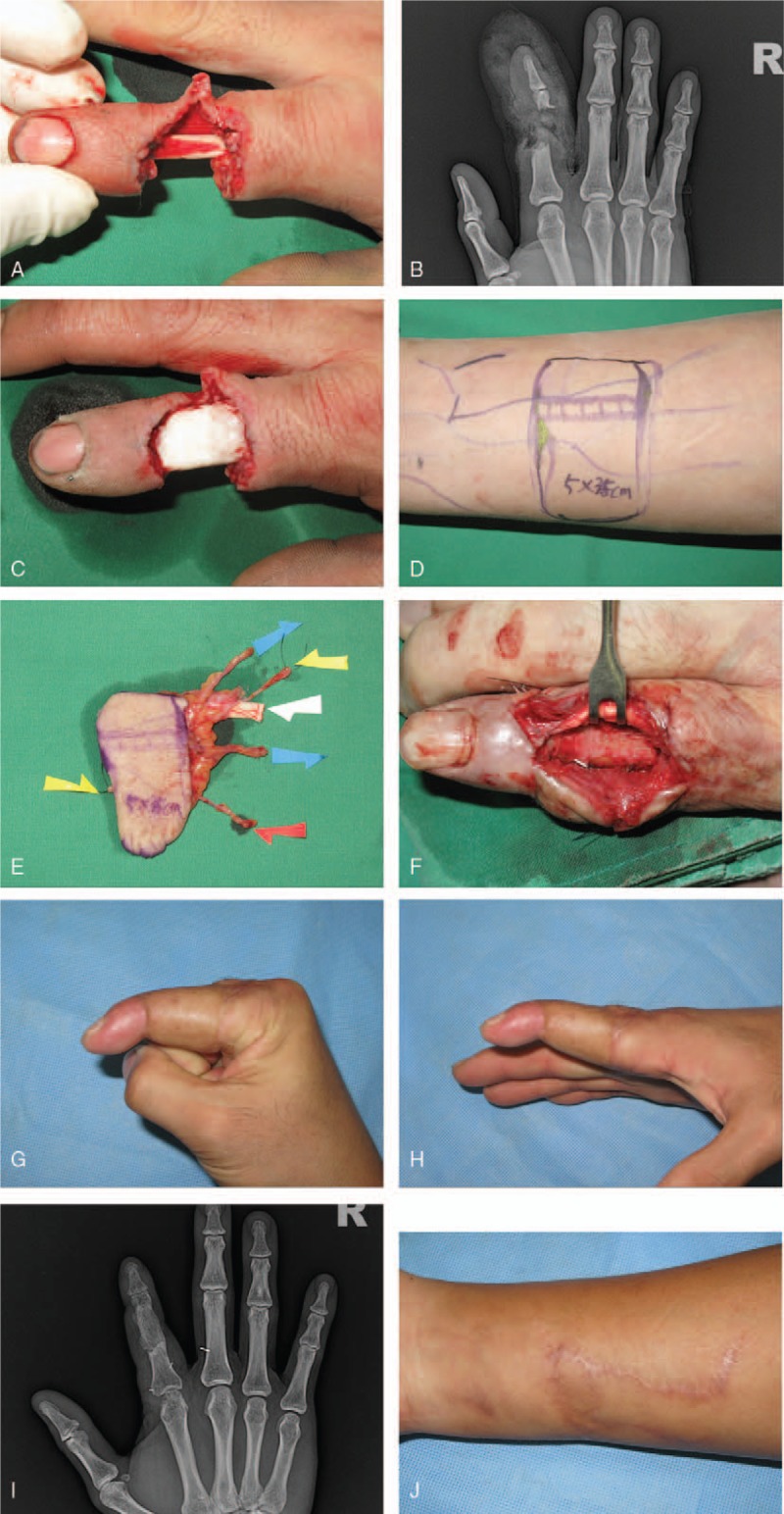
A 43-yr-old man with crush injury to the right IF. (A and B) Initial and preoperative views of right IF show compound defect on the proximal phalanx and middle phalanx area. (C) Bone cement in the right index finger. (D–E) Flap design on the ipsilateral volar aspect of the distal forearm and the dissected compound venous flap about 5 × 3.5 cm including 3 veins (blue arrows, efferent veins; red arrow, afferent vein for arterial repair), 1 cutaneous nerve (yellow arrows, for RDN reconstruction) and palmaris longus tendon (white arrow). (F) Three mo later, we removed previously inserted bone cement and did a bone graft with 2 × 1 cm sized autogenous corticocancellous bone from ilium. (G–J) Postoperative view of the donor and recipient site 8 mo later, showing that contour is almost normal and complete bone union. Active ROM is 80 degrees at the MP joint and 30 flexion contracture at the DIP joint. The static 2 PD is 14 mm. DIP = distal interphalangeal, IF = index fingers, MP = metacarpophalangeal, PD = point discrimination, RDN = radial digital nerve, ROM = range of motion.

**Figure 3:**
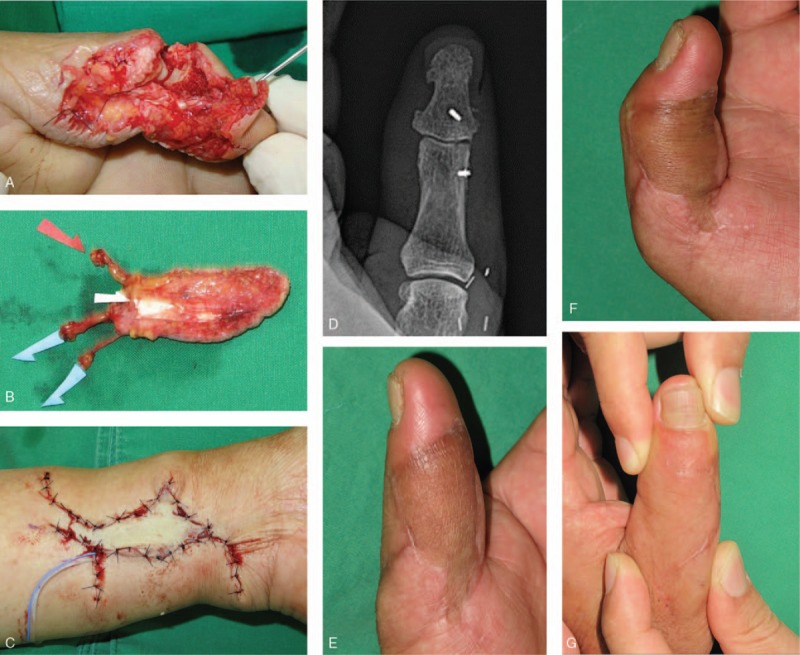
A 56-yr-old man with crush injury in left thumb. (A) Preoperative view of the left thumb shows severe crush injury on the soft tissue, partially comminuted fracture of the distal and proximal phalanges and radial CL defect. (B) Dissected compound venous flap about 3.5 × 3 cm including 2 veins and PL tendon. (C) Postoperative view of the donor site in the ipsilateral volar aspect of the distal forearm. (D–G) Postoperative views 5 mo later, showing that the active ROM of the IP joint is approximately 40 (0–40) degrees, with no arthritis and instability. CL = collateral ligament, IP = interphalangeal, PL = palmaris longus, ROM = range of motion.

When the flap was transferred to the recipient site, in CVF we sutured the skin first, then end-to-end anastomosis of the digital artery and afferent vein was carried out with 10-0 nylon (ETHILON 10-0 nylon suture; ETHICON, Cincinnati, OH) under the microscope (OPMY Vario/S88 system; Carl Zeiss Meditec, Germany) and then end-to-end anastomosis of the efferent vein and the dorsal vein of finger was carried out with the same method (Fig. 1). In IVF, first we repaired the nerve with 8-0 nylon under the microscope then we repaired the flap with the same method in CVF (Fig. 2). In TVF, the tendon was repaired first by using multiple figure-of-8 sutures or microsuture anchor (Micro Quick anchor plus (#4/0) suture; DePuy mitek, Raynham, MA) at the bone insertion site under appropriate tension and then the vessels were anastomosed (Fig. 3). The donor sites were usually closed primarily but in 9 (25.7%) cases, who had not been primary closure, were covered with skin graft.

### 2.4 Postoperative management and evaluation

Postoperatively the hand and forearm were wrapped in a bulky dressing and immobilized with above-elbow splint and the flap was monitored intensively for 7 days. Anticoagulation therapy with prostaglandin E1 (10 μg/d) and heparin (5000 unit/d) was administered for 1 week and aspirin 100 mg once daily for 1 month after surgery. Capillary refilling, surface temperature, color, and bulla formation of the flap were monitored.

Passive and active exercise of the metacarpophalangeal joint was started at 7 days after surgery unless necrosis was seen. In 2 (22%) cases with TVF without bone defect, rehabilitation was started on postoperative 2 weeks, using a dynamic splint with a volar block to restrict finger flexion. In 3 (8.6%) cases with surgical syndactyly, division of the flap was performed at 12 weeks after surgery.

Retrospectively, number of anastomosed vessels, size of the flap, and survival of flap, amount of necrosis, complications were investigated with the medical records of the patients. In addition, functional evaluation such as sensory recovery and range of motion (ROM) was assessed in patients who were available for follow-up after IVF or TVF transfer. Sensory recovery was evaluated with static 2 point discrimination (PD) and ROM was evaluated with goniometer.

### 2.5 Statistical analysis

IBM SPSS version 20.0 (IBM Corp, Armonk, NY) program was used for the statistical analysis of data. We used a Fisher exact test to compare the survival rate according to the vascularity of the recipient bed (good, more than 50% avascularity, partially devascularity at the distal part) excepting infected bed. When the *P* value was less than .05, it was considered statistically significant.^[9]^

## 3 Results

Average size of flap was 15.39 ± 6.24 cm^2^ (range, 3 × 2.5 to 8 × 3.5 cm). One afferent vessel was anastomosed in all cases. One efferent vein was anastomosed in 21 (60%) cases, 2 veins in 14 (40%) cases, accounting for an average of 1.4 ± 0.5 efferent veins.

Of the 35 cases, 29 (82.9%) cases (including 3 cases who had more than 50% avascularity recipient bed) showed complete survival. 3 (8.6%) cases, which had partially devascularity of distal phalanx in recipient bed, showed partial necrosis. Partial necrosis of less than 10% of the total area was successfully treated with secondary wound closure. One case of partial necrosis of 30% was treated with additional skin graft. There were statistically significant differences in survival rate according to the vascularity of the recipient bed (*P* = .015). Complete flap necrosis appeared in 2 cases who had infected recipient bed. There was no other specific complication.

In 9 cases of IVF, average static 2 PD was 10.5 ± 0.97 mm (range, 6–14 mm). In 3 (33.3%) cases among 9 cases of TVF, average active ROM of the proximal interphalangeal joint was 60 ± 34.64 degrees: 30 ± 17.32 degrees in the DIP joint and 40 degrees in the IP joint of thumb with no instability.

## 4 Discussion

An AVFs have a different hemodynamics compared to other conventional free flap.^[10,11]^ Arterialization of the venous system results in high-pressure blood flow through the venous system. On account of the increased blood pressure in the venous system, in the postoperative period, AVF can become edematous, discoloration and may progress to develop ecchymosis followed by superficial epidermolysis. There can be also difficulties in flap monitoring, low survival rate of large flap.^[1,12,13]^ For these characteristics of the flap, various techniques have been used to decrease high blood pressure in the venous system and to increase survival rate of flap. Woo et al^[7,14]^ stressed the importance of the number of draining vessels, vascular network within the flap, and the vascularity of recipient bed. Therefore, the clinical use of venous flaps for improving the survival rate should be ideal for acute, fresh, and partially avascular recipient wound coverage. However this use should be avoided in chronic, infected, and totally avascular recipient beds.

We experienced partial necrosis in 4 cases. In 1 case, partial necrosis occurred at the extended incision line within the flap, which had been made by author's mistake during the flap harvesting. In 3 cases among them, partial necrosis occurred at the area of less vein distribution within the flap, that is, it was the far distal area from the pedicles, which was contacted with periphery of the recipient bed with devascularity. Total necrosis was in 2 cases. In spite of the flap survival more than 1 week, total necrosis progress in 2 weeks due to continuous discharge between the flap and the recipient site. The reason is that the postoperative congestion was not resolved due to the failure of horizontal and vertical neovascularization between the flap and the recipient site.

We also extended the indications of flap, applied AVF in 3 cases, who had bone cement at the bone defect site. Considering the importance of recipient bed's vascularity which plays a critical role in flap survival, in these cases AVF could be so dangerous. But we could get the survival of flap with decrease of congestion via the periphery healing between the flap and recipient site.

When we reviewed the result mentioned above, there were statistically significant differences in survival rate according to the vascularity of the recipient bed. This suggests that the recipient bed vascularity is important. Among them peripheral vascularity is more important for complete survival of AVF; however, the sample size is too small to conclude this on a statistical basis. Venous flap should be also avoided in any ongoing infectious bed.

AVFs have advantages when 1 stage reconstruction is possible in composite tissue defect finger.^[7,15–18]^ Especially when the donor site is forearm, tendons, or nerves can be incorporated within the flap for reconstruction of composite defect. We have experienced in 9 cases reconstruction with a TVF with the PL tendon. We got the satisfactory results in all cases. Therefore we think that the indication of TVF can be extended to the reconstruction of CL and flexor tendon.

The medial and lateral antebrachial cutaneous nerve and its branched can also be identified and included in an IVF harvested from the distal third of the forearm. In 9 cases of reconstruction with an IVF for digital nerve and sensory of volar surface, we got 10.5 ± 0.97 mm mean static 2-PD. This result explains that we may decide an IVF which has an exact role in sensory reconstruction of fingers.

With the recent advances of the concept of perforator flap, finger reconstruction methods using micro-perforation flaps have been introduced.^[1–3]^ Unlike the venous flap, this is a conventional free flap that is hemodynamically more physiological, which makes it available for use regardless of the vascularity of the recipient bed. Among these flaps, free digital and free medial plantar artery perforator flaps are highly useful for sensation restoration of the fingertips and pulp defects while minimizing donor site morbidity.^[1,2]^ One drawback is that they require super microsurgical skill, where the entire surgery must be performed under an operating microscope in cases involving extremely small-sized diameter blood vessels. Furthermore, it is difficult to obtain medium to large flaps. By contrast, large free proximal peroneal artery perforator flaps are easier to obtain. Although they do not require super microsurgical skills, perforator dissection may be difficult. Moreover, 1-stage reconstruction for composite tissue defects is impossible with free proximal peroneal artery perforator flaps as opposed to venous flaps.

Donor site for AVFs have included the volar aspect of the forearm, the thenar and hypothenar area, the dorsum of the foot, and the medial aspect of calf.^[19]^ The influence of donor site on the survival of AVFs may be attributed to the configuration of venous network of different donor sites. The configuration of the dorsal skin of digits is more favorable than that of the volar aspect of the forearm, while the donor site of the lower leg, in which there is a poor venous network, is considered the last choice of venous flaps.^[20,21]^ So in case of hand reconstruction, the most common donor site is the volar aspect of the ipsilateral forearm. The forearm is the most common donor site for large flap. The thenar and hypothenar area are also appropriate for digit reconstruction. However, the durable glabrous skin from these area may be used for reconstruction of small-sized defects of the finger pulp.^[22]^ Therefore, we chose the distal forearm volar aspect as the donor site because of its similar venous network configuration with that of the digits, the availability of medium or large flaps, the availability of compound tissues such as nerves or tendons, the similarity to the skin texture, and the possibility of obtaining the flap from 1 operation field.

Our study has several limitations. First, our study was a retrospective observational study that could not be compared with the results of conventional free flaps for finger reconstruction. Second, we compared the survival rate according to the vascularity of the recipient bed, but only a few cases were analyzed. Third, only a few cases involving neurocutaneous and tendocutaneous flaps were included. For this reason, we could not present more-reliable data on sensory and ROM recovery. Fourth, we were unable to assess the level of return to daily living in patients during the last follow-up. Therefore, data on this could not be presented in the results. Fifth, the first author, who was the surgeon, processed and analyzed all the data.

## 5 Conclusion

AVF is a useful procedure for single-stage reconstruction of a soft tissue or combined defect of a finger. We consider that this procedure could be applied to finger, despite an avascular or hypovascular recipient bed if vascularity of the periphery of recipient bed is good and if the recipient beds are free from infection.

## Author contributions

**Data curation:** Malrey Lee, Young Keun Lee, Dong Hee Kim.

**Investigation:** Young Keun Lee.

**Methodology:** Malrey Lee, Young Keun Lee.

**Supervision:** Malrey Lee.

**Writing – original draft:** Malrey Lee, Young Keun Lee.

**Writing – review and editing:** Young Keun Lee.
